# The relationship of early expressed milk quantity and later full breastmilk feeding after very preterm birth: A cohort study

**DOI:** 10.1111/mcn.13719

**Published:** 2024-09-06

**Authors:** Ilana Levene, Frances O'Brien, Mary Fewtrell, Maria A. Quigley

**Affiliations:** ^1^ National Perinatal Epidemiology Unit, Oxford Population Health University of Oxford Oxford UK; ^2^ Newborn Care John Radcliffe Hospital Oxford UK; ^3^ UCL Great Ormond Street Institute of Child Health University College London London UK

**Keywords:** breast feeding, human, infant, intensive care units, lactation, milk, neonatal, newborn, premature birth, ROC curve

## Abstract

When infants cannot directly breastfeed after birth, mothers are advised to initiate lactation through mechanical expression. Families are recommended to target an expression volume of at least 500–750 mL by Day 14 after birth, as this is considered a ‘critical window’ to establish milk supply. This is challenging for many mothers after a very preterm birth. This article explores the relationship of early milk quantity and later full breastmilk feeding as a ‘gold standard’ outcome, using statistical techniques designed for diagnostic tests. A cohort of 132 mothers of infants born at 23 + 0 to 31 + 6 weeks' gestational age submitted expressing logs on Day 4, 14 and 21 after birth and provided later feeding outcome. Using receiver operating characteristic (ROC) analysis, the following 24‐h milk quantities were identified as associated with high probability of full breastmilk at 36 weeks' post‐menstrual age (PMA): on Day 4, ≥250 g (specificity 88%; positive predictive value 88%) and on Day 21 ≥650 g (specificity 88%; positive predictive value 91%). The following values were identified as associated with low probability of full breastmilk at 36 weeks' PMA: on Day 4 <50 g (sensitivity 92%; negative predictive value 72%) and on Day 21 <250 g (sensitivity 90%; negative predictive value 70%). Participants exceeding the high thresholds had 3–4 times increased likelihood of full breastmilk, whereas those below the low thresholds had 3–5 times lower likelihood. These thresholds have potential as targets for families, to provide individualised prognostic information and to help clinicians target more intensive lactation support.

## Key messages

- Mothers of very preterm infants who expressed at least 250 g at day four after birth had a high probability of providing later full breastmilk: 88% (95% CI: 72%–97%). This was similar for those who expressed at least 650 g at Day 21: 91% (95% CI: 77%–98%).
- Mothers of very preterm infants who expressed less than 50 g at day four after birth had a low probability of providing later full breastmilk: 28% (95% CI: 10%–53%). This was similar for those who expressed less than 250 g at Day 21: 30% (95% CI: 12%–54%).
- These thresholds may be useful for families to understand their lactation trajectories and for clinicians to target support.

## Graphical abstract

In this observational study, we identified a series of thresholds for early expressed milk quantity that risk stratified long‐term full breastmilk feeding after very preterm birth. These thresholds have potential as targets for families, to provide individualised prognostic information and to help clinicians direct more intensive lactation support.

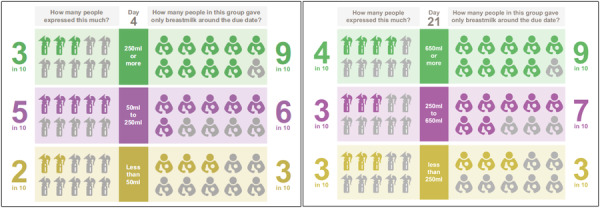

## 1 INTRODUCTION

Up to 18% of infants have medical needs requiring special care after birth, and this increases to 79% of preterm infants (Australian Institute of Health and Welfare, 2022). When infants are admitted to a neonatal unit, they are often unable to feed orally, particularly after very preterm birth (less than 32 weeks' postmenstrual age; PMA). Mother's own milk (MOM) is the optimal nutrition for all infants and has even more impact on those who are sick and preterm. In this context, MOM provides protection against conditions such as necrotising enterocolitis and retinopathy of prematurity and improves neurocognitive outcomes (Belfort et al., 2022; Corpeleijn et al., 2016; Vohr et al., 2007; Zhou et al., 2015). Increasing exclusive MOM feeding for very premature infants is included in the top 20 research priorities for premature birth by the World Health Organization, 2022.

In this setting, mothers mechanically express milk to provide nutrition for the infant, which can be challenging. Despite maternal motivation, there is a high risk of poor milk supply and nonexclusive MOM feeding (Akerström et al., 2007; Morag et al., 2016; Perrella et al., 2012). Milk volumes are a focus of anxiety for parents (Ikonen et al., 2016; Mӧrelius et al., 2020; Palmquist et al., 2020). In addition to providing milk, expression in the first weeks after birth also performs an important role in the establishment of long‐term milk supply. This is because of evidence of a ‘critical window’ to establish full lactation–if sufficient quantities are not expressed at this early period, there may be a ceiling of later supply (Hill et al., 2005a; Kent et al., 2016). Preterm infants have a very low milk requirement in the early days and weeks of life but may after some months have similar requirements to term infants.

In the UK, there is a recommendation to target at least 750 mL expressed milk yield per day by Day 10–14 after birth (UNICEF UK, 2013) because this is the average intake of a healthy, term infant at this stage (Kent et al., 2021; Kent, 1999). International recommendations mention that 720 mL in 24 h is a physiological norm and that expressing at least 500 mL in 24 h by Day 14 has good prognostic value (Maastrup et al., 2022; World Health Organization, 2022). The frequency of milk expression is also an important factor in determining expressed milk quantity (Bendixen et al., 2022).

Parents have unanswered questions on this topic, reporting that they don't know how much milk is considered adequate (Ikonen et al., 2016) and also asking about their individualised chances of long‐term lactation ‘success’ given their current milk yield. For example, during our preparatory parent involvement work (Levene, Alderdice, et al., 2022), when asked what questions they had about milk expression, responses included “What is the likelihood of… being able to exclusively breastfeed at full term?” “Guidance on how much milk should be produced through the first couple of weeks, and prompt identification of a woman who isn't producing enough,” “info about how much you should be expressing would be helpful. I never knew how much was a good amount and constantly worried” and “Depending on amounts expressed nurses should be able to give personalised advice.”^1^

Achievement of the recommendation to express at least 750 mL in 24 h in the NICU setting is relatively poor (Fewtrell et al., 2016; Patel et al., 2019). Although this may be the average intake for term infants, some healthy, thriving infants take in as little as 478 g per day at the breast (Kent et al., 2007). Robust evidence is therefore required on the benefit of specific yield targets in this setting. Data could not only direct parents' efforts but also give prognostic value and direct clinicians on which families might benefit from more intensive lactation support.

Existing observational studies, primarily related to preterm infants (Fewtrell et al., 2016; Gomez‐Juge et al., 2023; Hill & Aldag, 2005; Hill et al., 2005b, 2005a; Ru et al., 2020), have showed strong association of early milk yield (7–14 days after birth) and later milk yield. The threshold of 500 g in 24 h at Day 10–14 is associated with being more likely to receive any MOM at discharge (Hoban et al., 2018; Patel et al., 2019) and with 100% sensitivity (but only 67% specificity) for the outcome of expressing at least 500 g at week five (Hill et al., 1999). ROC analysis in a small study (Ru et al., 2020) identified 24‐h yield on Day 7 of 407 g and on Day 14 of 518 g as the values that maximised sensitivity and specificity when using an outcome of Day 42 milk yield of at least 750 g. It is notable that current evidence predominantly uses the parent‐focused, indirect outcome of later expressed milk yield, rather than the infant‐focused, direct outcome of long‐term MOM feeding (by direct breastfeeding or provision of MOM by an alternate route).

The aim of this study was to analyse the association of expressed milk yield in the first 3 weeks after very preterm birth with long‐term full MOM feeding outcomes. The objective was to derive yield quantities with good predictive value for later full MOM feeding. In this sense, early milk yield would function as a ‘diagnostic test’ for long‐term feeding outcome, using statistical techniques designed for diagnostic tests.

## 2 METHODS

### 2.1 Trial design

This analysis uses data collected for a randomised controlled trial. A detailed protocol has been published (Levene, Bell, et al., 2022). In brief, 132 people who had given birth to one or two infants between 23 + 0 and 31 + 6 weeks' PMA were recruited. The baseline questionnaire included intention to fully MOM feed around the time of discharge home. Ethnic background was self‐defined by participants in reference to UK Office for National Statistics categorisation (Office for National Statistics, 2022).

Participants were given a portable scale accurate to 0.1 g (Kabalo) and filled in logs each time they expressed milk for 24 h on three specific timepoints (Day 4, 14 and 21 after birth). The specific gravity of human milk is 1.03; so, 1 g and 1 mL can be considered near equivalent (Kumar et al., 2017).

Infant feeding status was assessed by parent text message at 36, 49 and 58 weeks' PMA (the latter referred to as two and 4 months' corrected age; CA). At 36 weeks' PMA, the data were extracted from infant medical notes if there was no parental response. Both the 2 months' and 4 months' CA assessments were only requested for participants in the first 10 months of recruitment for reasons of efficiency within a doctoral project timeline.

Participants were recruited in four tertiary and local neonatal units in the United Kingdom. Three of the four have UNICEF UK Baby Friendly Initiative level three accreditation. All units provide free hospital grade pumps both at the hospital and home and have dedicated infant feeding support staff.

The study was approved by the Bloomsbury Research Ethics Committee, London (21/LO/0279) and registered as ISRCTN 16356650. This exploratory analysis was pre‐specified in the registered protocol. The study was powered in relation to the randomised controlled trial's primary outcome, as described in the published protocol. Specifically, the study had 80% power to detect a difference in mean 24‐h milk quantity of 155 g between two categories of a binary variable.

### 2.2 Variable definitions

The outcome variable was full mother's own milk (MOM). Full MOM was defined as the provision of only MOM and no infant formula in the past 24 h. Complementary food was not considered in this definition, as the appropriate timing of the introduction of complementary food after very preterm birth is not well defined (King, 2009). Where a participant reported having stopped lactating at an earlier timepoint, they were assumed to have no MOM at any later timepoints. Note that this is not the same as the stricter definition of ‘exclusive MOM,’ which refers to lifetime exclusivity (i.e., provision of only MOM up to and including the timepoint in question).

Three timepoints for the outcome of full MOM were available for statistical analysis. However, 36 weeks' PMA was the only timepoint with sufficient ‘event’ numbers, and therefore, this was used as the main outcome timepoint. A requirement for at least 40 ‘events’ (full MOM provision and less than full MOM provision) was pre‐specified to facilitate the inclusion of 4–8 variables with a rule of 5–10 events per variable (Vittinghoff & McCulloch, 2007). Some results were also reported in relation to exclusive MOM at 2 months' CA, but this timepoint was not used as an outcome in statistical models.

The key explanatory variables were 24‐h milk quantity on Day 4, 14 and 21 after birth. These variables were analysed as both continuous and binary, using cut‐offs defined by previous literature or clinical guidance (500 g and 750 g, respectively).

Lactogenesis II (secretory activation) was also used as an explanatory variable related to early milk quantity. Secretory activation is the onset of copious milk production and is delayed if it has not occurred by Day 4 after birth (Brownell et al., 2012). Participants who had documented expressing at least 20 g of milk for two consecutive sessions at Day 4 were considered to have achieved lactogenesis II. For mothers who have given birth very preterm, this may be the most accurate definition because they more rarely have a feeling of breast fullness or biochemical changes within the milk (Parker et al., 2021). As a pragmatic measure where only one expressing session was logged, lactogenesis II was assumed to have been achieved if the total quantity expressed at that session was more than 40 g.

Baseline demographic and perinatal variables were included as potential confounding variables, as well as frequency of milk expression. These were identified as potentially important from the literature. The index of multiple deprivation quintile was derived from participant postcode–this measure is assigned by the UK government according to multiple measures of deprivation.

### 2.3 Statistical analysis

Analysis took place on a complete case basis. Binomial 95% confidence intervals were calculated for full MOM prevalence.

Analysis of full MOM used Poisson regression with robust standard errors to estimate risk ratios. In multivariable analysis, each type of early milk quantity explanatory variable was analysed in a separate model, and adjustment was made for potential confounders. Potential confounders were included in multivariable analysis if they showed association with the outcome in univariable analysis with *p* < 0.2.

To identify the pattern of association between early milk quantity and later full MOM, ROC analysis with exact binomial confidence intervals was performed. Area under the curve (AUC) of more than 0.70 was considered acceptable accuracy (Swets, 1988). It was expected that the variables studied would not demonstrate a single effective cut‐point with both high sensitivity and specificity but rather a ‘three‐zone’ division (Feinstein, 1990) with a central ‘grey zone’ (Coste & Pouchot, 2003). Three‐zone cut‐points are derived with reference to test characteristics and the ‘costs’ of false positives and negatives (Coste & Pouchot, 2003; Feinstein, 1990). The lowest yield cut‐point with specificity of at least 80% was identified as a potential ‘rule in’ test, meaning it would have a high association with positive feeding outcome. The highest yield cut‐point with sensitivity of at least 90% was identified as a potential ‘rule out’ test, meaning it would have a high association with negative feeding outcome. Maximisation of the likelihood ratio was also considered for the ‘rule in’ threshold.

## 3 RESULTS

### 3.1 Participant flow

Supporting Information S1: Figure S1 demonstrates the number of participants providing data at each timepoint used in this analysis. Six participants could not be included in the analysis due to infant death before 36 weeks' PMA. At least one 24‐h expressing record was submitted by 108 participants and 80 participants submitted all three expressing records. Most of the missing milk expression data were from participants who chose to remain in the study but did not submit a valid milk expression record on Day 14 or 21. Seven participants chose to withdraw from the study during the milk expression stage. Those who gave a reason for withdrawal predominantly cited a lack of time/feeling overwhelmed.

### 3.2 Baseline characteristics

Table 1 shows the baseline characteristics of the cohort. Overall, 15% had twins, 60% were primiparous and 70% planned to fully MOM feed. The cohort was sociodemographically diverse; 18% of participants were Black and 18% Asian, 38% lived in the most deprived two quintiles of England and 32% left full time education before 19 years of age. Mean maternal age was 32.8 ± 6.3 years, and gestation at birth was 27.8 ± 2.4 weeks' PMA.

**Table 1:** Baseline characteristics of the cohort.

	Whole cohort *n* = 132 (*n*, %)	Missing data^†^
Ethnic background		12
*Asian*	22 (18.3%)	
*Black*	21 (17.5%)	
*White*	72 (60%)	
*Other*	5 (4.2%)	
Index of multiple deprivation quintile		1
*1 (most deprived)*	24 (18.3%)	
*2*	26 (19.8%)	
*3*	22 (16.8%)	
*4*	31 (23.7%)	
*5 (least deprived)*	28 (21.4%)	
*Age*		0
*<25 years*	15 (11.4%)	
*25 to* <*30 years*	26 (19.7%)	
*30 to <35 years*	54 (40.9%)	
*≥35 years*	37 (28.0%)	
Age at ceasing full time education		12
*16 years or less*	14 (11.7%)	
*17–18 years*	24 (20%)	
*19 years* or more	82 (68.3%)	
Presence of partner	108 (89.3%)	11
Smoker	8 (6.6%)	11
Caesarean birth	75 (56.8%)	0
Multiple birth	20 (15.2%)	0
Primiparous	74 (59.7%)	8
Gestation at birth		0
*23 to <26 weeks*	34 (25.8%)	
*26 to <28* weeks	37 (28%)	
*28 to <30 weeks*	29 (22%)	
*30 to <32 weeks*	32 (24.2%)	
Infant/s ventilated at baseline	47 (35.6%)	0
Baseline intention to fully breastmilk feed	85 (70.2%)	11
Longest length of prior breastmilk feeding (multipara only)		6
*<26 weeks (6 months)*	18/44 (40.9%)	
Time to first expression after birth		12
*≤6 h*	62 (51.7%)	

*Note*: ^†^Missing data not included in percentage denominators.

Abbreviation: MOM, mother's own milk.

### 3.3 Description of lactation outcomes

Daily milk quantity increased over time from a median of 155 g (IQR: 66–296, *n* = 101) on Day 4 to 490 g (IQR: 248–830, *n* = 90) on Day 21. Delayed lactogenesis II was identified in 31.7% on Day 4 (32/101). Milk yield on Day 14 and Day 21 showed very strong correlation (*r* = 0.90). Participants with twins had median milk quantity of 231 g on Day 4 and 618 g on Day 21.

Expressing frequency was median 5 (IQR: 4–7) on Day 4 and 6 (IQR: 5–7) on Day 21. Participants with twins had a median expressing frequency of 6 on Day 4 and 6.5 on Day 21.

In the whole population, 64.9% (74/114) were fully MOM feeding at 36 weeks' PMA and 86.0% were giving any MOM (98/114). For participants with twins, these figures were 64.7% (11/17) and 94.1% (16/17), respectively. Supporting Information S1: Figure S2 shows the trajectory of feeding outcomes over time for the participants recruited into the complete follow up phase. By 4 months' CA, full MOM had decreased to 14.0% (8/57) and any MOM to 38.6% (22/57). Infants were an average of 8 weeks, 4.6 months and 6.7 months' actual age at the three timepoints, respectively.

In the complete follow‐up phase, there was no evidence of a difference in either baseline characteristics or full MOM feeding at 36 weeks' PMA between participants with complete and incomplete data for the three feeding status timepoints.

### 3.4 Determinants of full MOM at 36 weeks' PMA

Table 2 shows the relationship of each milk quantity variable with full MOM at 36 weeks' PMA, along with theoretically important baseline variables and expressing frequency. Expressed milk yield on Day 4, 14 and 21 were all associated with full MOM, with a 6%–11% increased risk of full MOM per 100 g milk (aRR of 1.06 and 1.11). Lactogenesis II achievement at day 4 was also highly associated with the outcome, with aRR of 2.8 (95% CI: 1.5–5.3).

**Table 2:** Crude and adjusted relative risk for full mother's own milk feeding at 36 weeks' postmenstrual age.

	Unadjusted RR (95% CI)	*p*	Adjusted RR (95% CI)	*p*
Milk quantity variables				
Lactogenesis II achieved at Day 4	2.71 (1.49–4.92)	0.001	2.85 (1.53–5.33)	0.001
Day 4 yield (per 100 g)	1.11 (1.04–1.19)	0.001	1.10 (1.03–1.17)	0.004
Day 14 yield (per 100 g)	1.07 (1.01–1.13)	0.02	1.06 (1.00–1.12)	0.03
Day 21 yield (per 100 g)	1.06 (1.01–1.12)	0.01	1.06 (1.01–1.12)	0.01
Day 14 yield ≥500 g	1.75 (1.25–2.44)	0.001	1.64 (1.17–2.29)	0.004
Day 21 yield ≥500 g	1.64 (1.19–2.26)	0.003	1.61 (1.19–2.19)	0.002
Day 14 yield ≥750 g	1.34 (1.01–1.78)	0.04	1.24 (0.94–1.64)	0.13
Day 21 yield ≥750 g	1.46 (1.14–1.88)	0.003	1.50 (1.15–1.96)	0.003
Milk expression variables				
Day 4 frequency (sessions per day)	1.02 (0.96–1.09)	0.53		
Day 14 frequency (sessions per day)	1.02 (0.93–1.11)	0.68		
Day 21 frequency (sessions per day)	1.05 (0.97–1.14)	0.26		
Baseline variables (perinatal)				
Multiple birth	1.00 (0.68–1.46)	0.99		
Caesarean birth^a^	0.83 (0.63–1.07)	0.15		
Primiparous	0.99 (0.75–1.30)	0.94		
Infant/s ventilated at baseline^a^	0.69 (0.49–0.98)	0.04		
Gestational age at birth (per week)	1.04 (0.98–1.10)	0.23		
Baseline intention to fullly breastmilk feed^a^	1.32 (0.92–1.91)	0.14		
Presence of partner^a^	1.90 (0.86–4.21)	0.11		
Baseline variables (demographic)				
Maternal age (per 10 years)	0.86 (0.67–1.12)	0.26		
Left full time education >18 years	1.15 (0.82–1.59)	0.42		
Ethnic background				
*White*	1			
*Asian*	1.24 (0.92–1.68)	0.15		
*Black*	0.79 (0.49–1.27)	0.33		
Index of multiple deprivation quintile				
*5 (least deprived)*	1			
*4*	1.27 (0.86–1.87)	0.24		
*3*	1.21 (0.80–1.83)	0.36		
*2*	0.83 (0.49–1.41)	0.50		
*1 (most deprived)*	1.05 (0.66–1.69)	0.83		

Abbreviations: MOM, mother's own milk; RR, relative risk.

^a^ Adjustment variables. Adjusted analysis also included the randomised allocation. Each adjusted relative risk is the result of a separate model, all of which were adjusted for the same covariates.

The international recommendation for milk yield (500 g in 24 h) was associated with full MOM when measured at both day 14 and 21. The UK recommendation (750 g in 24 h) was associated with full MOM when measured at Day 14 or Day 21 in unadjusted analysis but only when measured at Day 21 in adjusted analysis. Adjustment for potentially important confounders made minimal difference to other relationships. Participants with multiple birth were no more or less likely to provide full MOM than those with singleton birth.

Using the outcome of full MOM at 36 weeks' PMA in ROC analysis, the AUC was similar for milk yield on day 4, 14 and day 21: 0.79 (95% CI: 0.69–0.86), 0.78 (95% CI: 0.67–0.86) and 0.80 (95% CI: 0.69–0.88), respectively, all of which exceeded the ‘adequate’ threshold. These graphs are shown in Supporting Information S1: Figure S3. Adding other variables such as MOM feeding intention, multiple birth, expressing frequency and the randomised allocation did not improve the AUC, and therefore, these variables were not included in further analysis. Because of the high correlation of milk yield at Day 14 and 21, further analysis examined Day 4 and 21 milk yield only.

### 3.5 Overview of the “three‐zone” distribution derived

The derivation analysis described in more detail below results in the division of expressed milk quantity into three zones at both Day 4 and Day 21, which were associated with low, medium and high prevalence of later full MOM.

**Figure 1:**
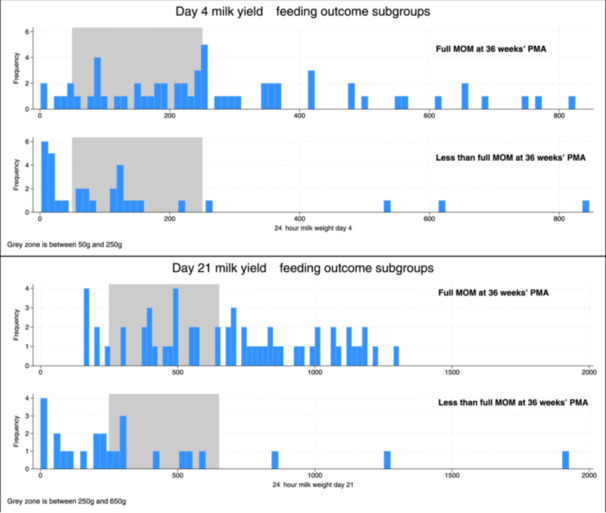
Histogram of milk yield at Day 4 and 21 by full mother's own milk at 36 weeks' postmenstrual age.

Table 3 shows the outcomes associated with these proposed yield thresholds, and Figure 1 demonstrates the distribution of participants' milk yield according to feeding outcome. Infographics suitable for parents are provided in Supporting Information S1: Figure S4 and S5. The following sections describe how the three zone values were derived.

**Table 3:** Feeding outcomes for proposed “three‐zone” categories of Day 4 and Day 21 milk yield.

	Full MOM at 36 weeks' PMA		Full MOM at two months' CA	
	*n*/*N* (%)	95% CI	*n*/*N* (%)	95% CI
Day 4 expressed milk yield in 24‐h				
<50 g	5/18 (28%)	10%–53%	1/14 (7%)	0%–34%
50 g to <250 g	27/42 (64%)	48%–78%	3/26 (12%)	2%–30%
≥250 g	29/33 (88%)	72%–97%	6/14 (43%)	18%–71%
Day 21 expressed milk yield in 24‐h				
<250 g	6/20 (30%)	12%–54%	1/13 (8%)	0%–36%
250 to <650 g	20/28 (71%)	51%–87%	2/17 (12%)	1%–36%
≥650 g	32/35 (91%)	77%–98%	8/16 (50%)	25%–75%

Abbreviations: CA, corrected age; CI, confidence interval; MOM, mother's own milk; PMA, postmenstrual age.

### 3.6 Deriving a high specificity (‘rule in’) threshold, Day 4

The lowest milk yield at Day 4 with more than 80% specificity for full MOM at 36 weeks' PMA was 149 g. Maximal positive likelihood ratio showed a double peak with high points at 175 g and 224 g (likelihood ratio 4.5 and 4.7 respectively). Diagnostic test criteria for these values were compared (rounded to the nearest 50 g; Supporting Information S1: Table S1), and 250 g was selected as the optimal ‘rule in’ threshold.

For those expressing more than 250 g at Day 4, the positive predictive values were 88% (29/33) and 43% (6/14) for full MOM at 36 weeks' PMA and 2 months' CA, respectively. Expressing more than 250 g at Day was associated with 3.8 and 3.3 times greater likelihood of these outcomes, respectively. 33% of participants expressed 250 g or more at day 4 (33/101).

### 3.7 Deriving a high sensitivity (‘rule out’) threshold, day 4

The highest milk yield at Day 4 with more than 90% sensitivity for full MOM at 36 weeks' PMA was 59 g. Test characteristics for this value were compared with thresholds associated with poor lactation outcomes in previous studies (140 and 153 mL), along with lactogenesis II status at Day 4 (Supporting Information S1: Table S2). The optimal ‘rule out’ threshold was selected as 50 g. Lactogenesis II also had high predictive value.

For those expressing less than 50 g at Day 4, the negative predictive values were 72% (13/18) and 93% (13/14) for full MOM at 36 weeks' PMA and 2 months’ CA, respectively. Expressing less than 50 g at Day 4 was associated with one fifth and one‐third the likelihood of these outcomes, respectively. 20% of participants expressed less than 50 g at Day 4 (20/101).

### 3.8 Deriving a high specificity (‘rule in’) threshold, Day 21

The lowest milk yield at day 21 with more than 80% specificity for full MOM at 36 weeks' PMA was 547 g, and the point of maximal positive likelihood ratio was 637.9 g (likelihood ratio 4.7). Diagnostic test criteria for these values (rounded to the nearest 50 g) were compared with existing clinical recommendations of 500 g and 750 g (Supporting Information S1: Table S1), and 650 g was selected as the optimal ‘rule in’ threshold.

For those expressing more than 650 g at day 21, the positive predictive values were 91% (32/35) and 50% (8/16) for full MOM at 36 weeks' PMA and 2 months' CA, respectively. Expressing more than 650 g at Day 21 was associated with 4.6 and 4.2 greater likelihood of these outcomes, respectively. 39% of participants expressed 650 g or more at day 21 (35/90).

### 3.9 Deriving a high sensitivity (‘rule out’) threshold, day 21

The highest milk yield at Day 21 with more than 90% sensitivity for full MOM at 36 weeks' PMA was 211 g. Diagnostic test criteria for the nearest rounded values were compared (Supporting Information S1: Table S1). In this population, 200 g and 250 g had similar test characteristics, and 250 g was selected as the optimal ‘rule out’ threshold.

For those expressing less than 250 g at Day 21, the negative predictive values were 70% (14/20) and 90% (36/40) for full MOM at 36 weeks' PMA and 2 months; CA, respectively. Expressing less than 250 g at Day 21 was associated with one fifth and one‐third the likelihood of these outcomes, respectively. 25% of participants expressed less than 250 g at Day 21 (23/90).

### 3.10 Participant perception of milk supply

Participant perception of the sufficiency of their milk supply was more accurate in relation to these thresholds on Day 21 compared to Day 4. At Day 4, 44% of those expressing **less than** 50 g in 24 h perceived they had a **good** milk supply. Similarly, 48% of those with delayed lactogenesis II at day 4 perceived they had a good milk supply. In contrast at day 21, 24% of participants expressing less than 250 g in 24 h perceived they had a good milk supply. Supporting Information S1: Figure S6 demonstrates the distribution of expressed yield by maternal perception of milk supply at each timepoint in relation to the proposed thresholds.

### 3.11 Movement between yield zones

**Figure 2:**
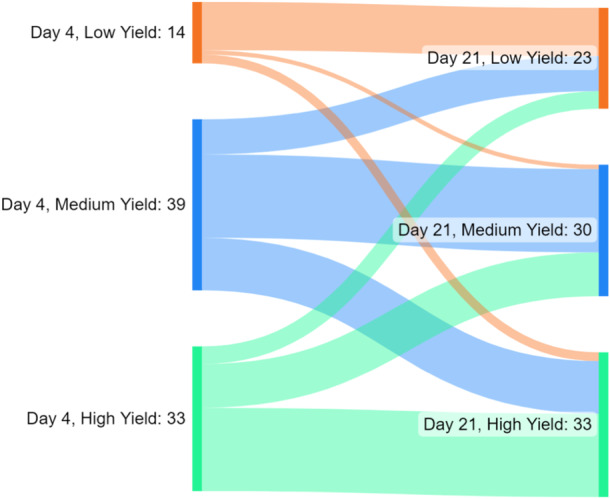
Sankey diagram showing the flow of participants between yield threshold groupings at Day 4 and Day 21.

Figure 2 shows the movement of participants between yield zones from Day 4 to Day 21. The two categories of particular clinical interest are 14 out of 33 participants with a high yield at Day 4 (>250 g) who move to a lower group by Day 21 (<650 g) and the 14 out of 53 participants who do not have high yield at Day 4 (<250 g) who moved to the high yield group by Day 21 (>650 g).

Variables associated with movement into the high yield category by day 21 (positive clinical outcome) were increased skin‐to‐skin contact on Day 14 (RR 1.3 per hour of skin‐to‐skin contact, 95% CI: 1.1–1.6; *p* = 0.001) and increased expressing frequency on day 21 (RR 1.3 per increment, 95% CI: 1.0–1.6; *p* = 0.02). In adjusted analysis, including both variables, only skin‐to‐skin contact duration at Day 14 remained significantly associated.

Variables associated with movement out of the high yield category by day 21 (negative clinical outcome) were lower expressing frequency on Day 4 (RR 0.7 per increment, 95% CI: 0.5–0.9; *p* = 0.001), lower expressing duration on Day 4 (RR: 0.7 per 30 min, 95% CI: 0.6–0.9; *p* = 0.002), longer maximal gap between expressions on Day 4 (RR: 1.1 per hour, 95% CI: 1.1–1.2; *p* = 0.001) and younger age (RR: 0.4 per 10 years, 95% CI: 0.2–0.8; *p* = 0.01). In adjusted analysis, including all these variables, only expressing frequency on Day 4 remained significantly associated.

## 4 DISCUSSION

### 4.1 Summary of findings

This analysis identified milk yield in the first 3 weeks after very preterm birth as closely associated with long‐term full MOM, which facilitated the proposal of milk yield categorisation as potential predictors for later lactation outcome. The “three‐zone” yield division successfully divided this sample population into those with low, intermediate and high likelihood of later full MOM. Participants expressing more than 250 g in 24 h at Day 4, or 650 g at Day 21, had 3–4 times increased likelihood of a full MOM, whereas those expressing less than 50 g in 24 h at Day 4, or 250 g at Day 21, had 3–5 times lower likelihood. In the middle “grey zone” group, pretest and posttest probabilities for full MOM were similar; so, expressed milk yield did not add new information to the likelihood of full MOM. It is common in clinical practice to use separate ‘rule in’ and ‘rule out’ thresholds in this way (Martín‐Rodríguez et al., 2021; National Institute for Health and Care Excellence, 2022).

While Day 4 yield showed a high level of association with feeding outcomes, some participants who did not have high yield at Day 4 moved into the high yield group by Day 21, which is a desirable outcome. The key variable associated with this movement was skin‐to‐skin contact duration, which is an important finding for staff and families experiencing early challenges to lactation. In contrast, the variable independently associated with movement from high to lower yield groups after Day 4 was expressing frequency at Day 4 rather than any later behaviour, which reinforces the need for frequent expressing even for those who are expressing good volumes in the first days after birth.

### 4.2 Placing the findings in context

Expressed milk yield was broadly similar in this study to previous cohorts (Bendixen et al., 2022; Héon et al., 2016; Murase et al., 2014), although the frequency of delayed lactogenesis II is lower here than some (Bendixen et al., 2022; Dong et al., 2022; Ru et al., 2020). The sample is not representative of very preterm infants in general in the UK–it was predominantly recruited from Southern English units, which tend to have higher prevalence of MOM feeding than other regions (National Neonatal Audit Programme, 2022). This likely explains why the prevalence of any MOM at 36 weeks' PMA in this study (86%) was higher than the national prevalence at discharge home in the UK National Neonatal Audit Programme (60% in 2020; National Neonatal Audit Programme, 2021).

The prevalence of long‐term MOM reported here is lower than for very preterm infants in China (Dong et al., 2022) and Sweden (Åkerström et al., 2007) but higher than in Portugal (Rodrigues et al., 2018) and several other European countries (Bonnet et al., 2019). This is as expected given the national variation in breastfeeding rates for all infants, which are highly correlated with MOM outcome variability in very preterm infants (Bonet et al., 2011).

While many previous studies have shown that milk yield for mothers of preterm infants in the first weeks of life is associated with later milk yield (Fewtrell et al., 2016; Gomez‐Juge et al., 2023; Ru et al., 2020), only one has reported association of yield (at Day 4) with full MOM at discharge (Murase et al., 2014). The association of delayed lactogenesis II with poorer MOM outcome has been noted previously (Brownell et al., 2012; Dong et al., 2022; Yu et al., 2019). The findings here can't be directly compared to the limited number of previous studies examining test characteristics of yield thresholds as they refer to different timing (24‐h milk quantity of 407 g on Day 7, 518 g on Day 14 and 500 g on Day 14; Hill et al., 1999; Ru et al., 2020). These preceding studies were much smaller than the current report, which puts them at higher risk of bias. By using outcomes related to milk yield rather than MOM feeding, they also provided more indirect evidence than the current study.

The findings of the current study may not be generalisable to mothers of term infants who are expressing MOM due to infant illness, difficulties with breastfeeding or personal choice because of the higher early milk intake requirement of term infants.

The association of skin‐to‐skin contact with lactation outcomes is well established (Heller et al., 2021; Parker et al., 2019), with one study suggesting that every minute of skin‐to‐skin contact was associated with an increase of 2.5 mL (±1.2 mL) of expressed milk (Daniels et al., 2023). However, the result seen here that increased skin‐to‐skin contact is associated with a movement from a less optimal milk quantity to a more optimal milk quantity over time has not to our knowledge been shown before.

It was notable that multiple births did not change the results of the analysis, despite more milk being required to fully MOM feed twins. This may have been because there were a relatively small number of participants with twins. However, it would also be consistent with the concept that when sufficient milk is expressed in the early ‘critical window’, milk supply is able to respond to major changes in later demand, including the larger quantities needed to fully MOM feed twins near term.

### 4.3 Strengths and limitations

This proposal of a series of yield thresholds with predictive value for MOM feeding outcome is a unique contribution to the literature. The attempt to define ‘rule out’ thresholds marking high risk of less than full MOM targets parents' expressed desire for personalised prognostic information (Levene, Alderdice, et al., 2022), as well as potentially helping clinicians to intensify lactation support–particularly at the early stage of Day 4. The Day 4 thresholds may be particularly useful because mothers' existing understanding of what constitutes ‘good’ milk yield was noted to be worse at Day 4, with nearly half of participants expressing less than 50 g per day at Day 4 perceiving that they had a good milk yield. This implies a deficit in appropriate counselling for parents and possibly in staff understanding of appropriate milk yield in the days after birth.

This is a diverse cohort of mothers of very and extremely preterm infants, in terms of gestation, ethnic background, education and markers of deprivation. The prospective nature of the cohort is appropriate (Moons et al., 2009) for attempts to derive a prognostic model. The high level of intention to MOM feed may limit the generalisability of the results but also makes this sample a good experimental model to observe associations between expressing parameters and feeding outcomes. A key weakness is the sample size (Moons et al., 2009). Validation in a second, larger sample is desirable (Moons et al., 2009) before the thresholds proposed here are recommended clinically (although it should be noted that current clinical recommendations are based on poorer quality evidence than this study provides). Because of the increased loss to follow‐up at Day 21, the findings at day 4 likely have a lower risk of bias than those at Day 21.

There is also a potential problem in using full MOM at 36 weeks' PMA as the outcome in ROC analysis. If there had been sufficient numbers, we would have used full MOM at 4 months' corrected age to derive the yield thresholds because it is near the point of likely maximal milk demand (around the time of introduction of complementary feeds, which is recommended by a mixture of actual and corrected age in very preterm infants, along with developmental readiness [King, 2009]). It is possible that infants fully MOM feeding at 36 weeks' PMA could not continue for as long as their parents would have liked because a ceiling of milk supply was exposed later as demand increased over time. The evaluation of test characteristics derived from full MOM at 36 weeks' with the same outcome at 2 months' CA did not significantly change the threshold conclusions, which is reassuring. However, the small numbers fully MOM feeding at later time points, and the increased loss to follow‐up at 2 months' CA limits the robustness of this conclusion.

A final limitation to this study is the reliance on participants to weigh and log their expressed milk, which may have affected the accuracy of the data and also may have contributed to the loss to follow‐up. It is likely that participants who did not provide data had lower milk yield than those who did. The restriction to measurements of milk yield on three specific timepoints also limited the potential outcomes of analysis. However, the study design was explicitly recommended by parent collaborators to minimise parent burden, to ensure no interference with the immediate use of expressed milk and to reduce feelings of stigma through close interactions with research staff in the measurement of milk (Levene, Alderdice, et al., 2022).

## 5 CONCLUSIONS

This study has produced clinically directed yield thresholds for the first few weeks after very preterm birth, using long‐term full MOM as a gold standard outcome. These thresholds can be tested in future research for goal setting, prognostic information and potentially for targeting of lactation support.

## AUTHOR CONTRIBUTIONS

Ilana Levene conceived the study, drafted the protocol and supporting documents, managed the trial, recruited many of the participants, performed the analysis and drafted the manuscript. Mary Fewtrell, Maria A. Quigley and Frances O'Brien are Ilana Levene's doctoral supervisors; they supported the conception, preparation, management and analysis. All authors read and approved the final manuscript.

## CONFLICT OF INTEREST STATEMENT

MF receives an unrestricted research donation from Philips for research in infant nutrition, unrelated to this work. IL was previously on the working group for the British Association of Perinatal Medicine Quality Improvement Toolkit on perinatal optimisation of breastmilk.

## Supporting information

Supporting information.

## Data Availability

Deidentified individual participant data (including data dictionaries) may be made available. A review of all requests for sharing of the study data will take place as described in the National Perinatal Epidemiology Unit Standard Operating Procedures on data sharing. If agreed, any sharing of the data collected during the study must be in accordance to the Nuffield Department of Population Health and University of Oxford policies. Instructions on requesting access are provided here: https://www.npeu.ox.ac.uk/ctu/data-sharing.

## Footnotes

1 These specific quotes are not reported in the published paper but the methods with which they were obtained and the ethical approval for their publication are described there.
