# Supplementary material for: The relationship of early expressed milk quantity and later full breastmilk feeding after very preterm birth: A cohort study
Source: Matern Child Nutr. 2024 Sep 6;21(1):e13719. doi: 10.1111/mcn.13719 (PMC11650023; doi:10.1111/mcn.13719)
Supplement: Supplementary file 1 — Supporting information. [file MCN-21-e13719-s001.pdf]

Supplementary Material

Supplementary Figure 1: Participant data flow through the study

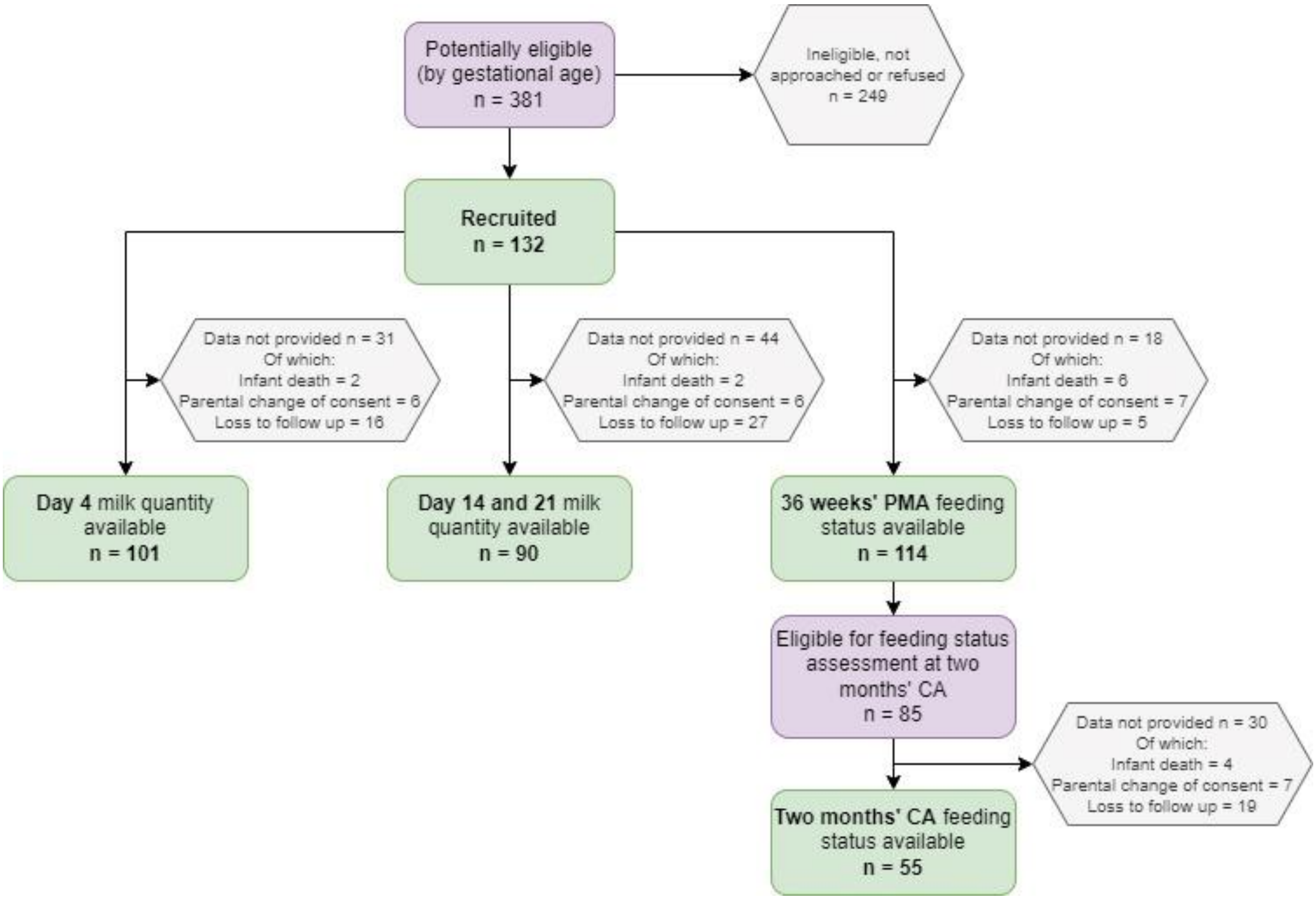

PMA = post-menstrual age. CA = corrected age

Supplementary Figure 2: Breastmilk feeding rate over time, in participants eligible for complete follow up only

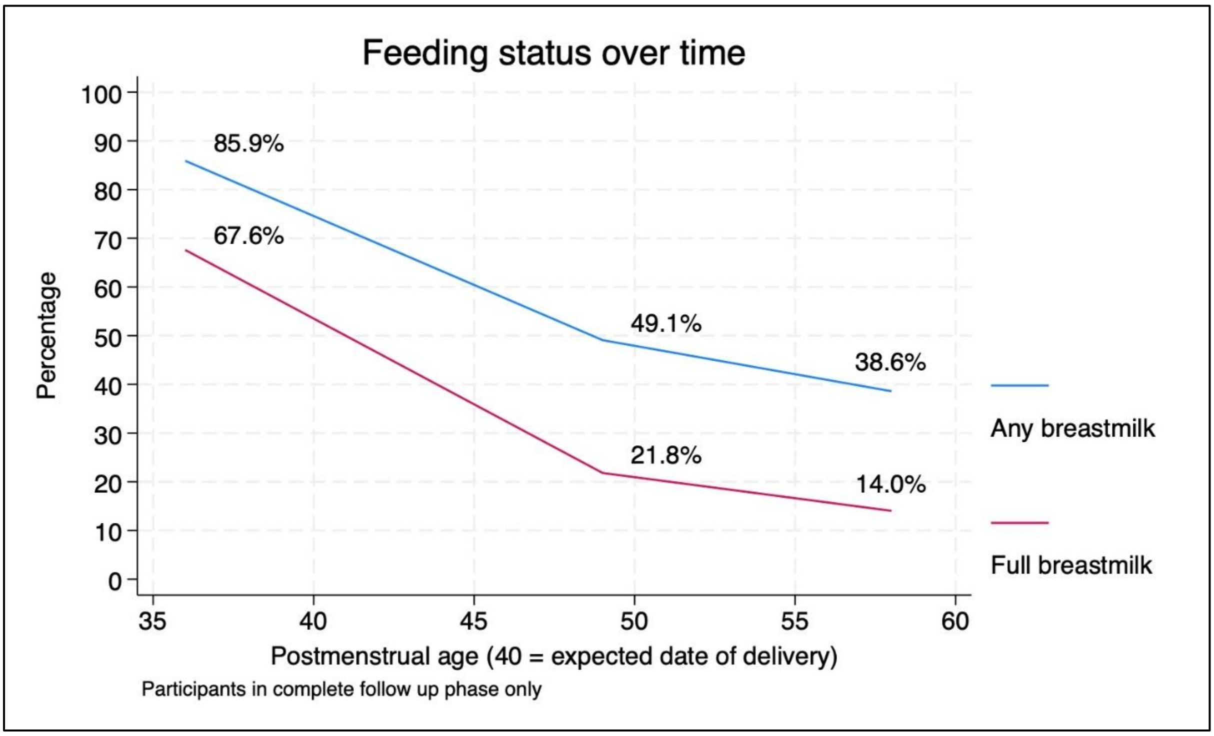

Supplementary Figure 3: Receiver Operating Characteristic (ROC) analysis using expressed milk yield at day 4, 14 and day 21 with an outcome of full MOM at 36 weeks' PMA

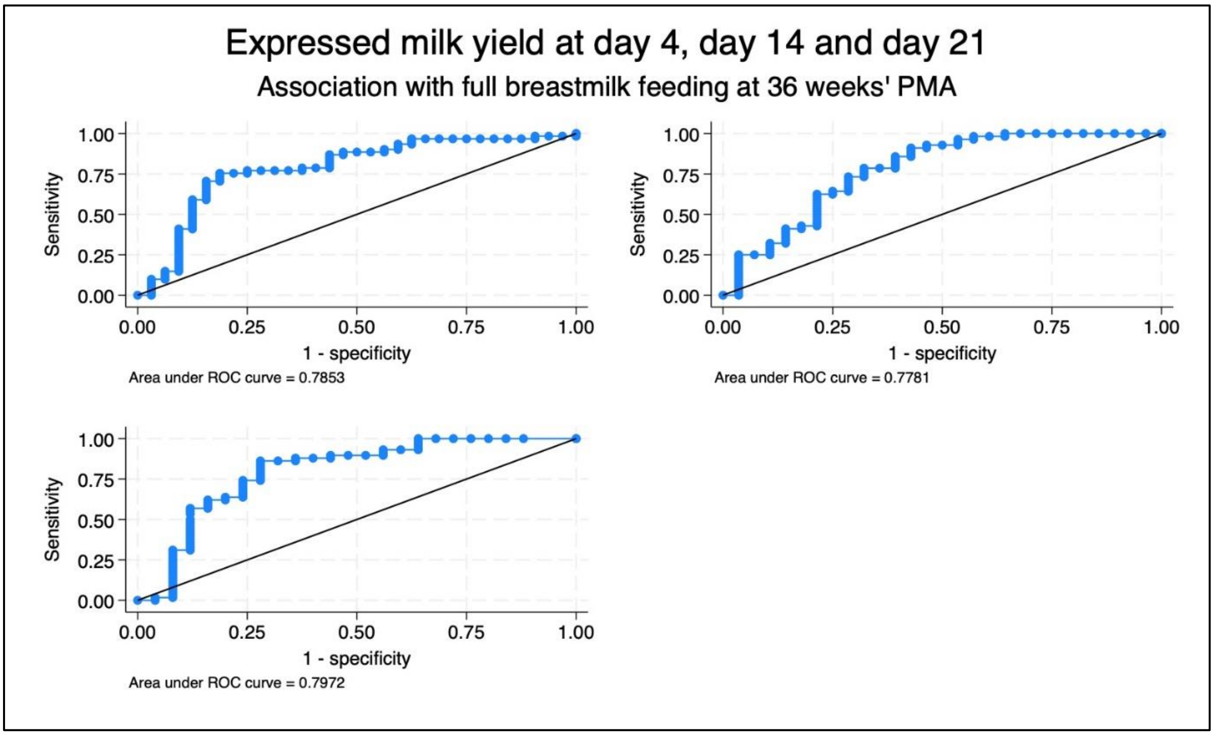

Supplementary Figure 4: Infographic for parents (day 4)

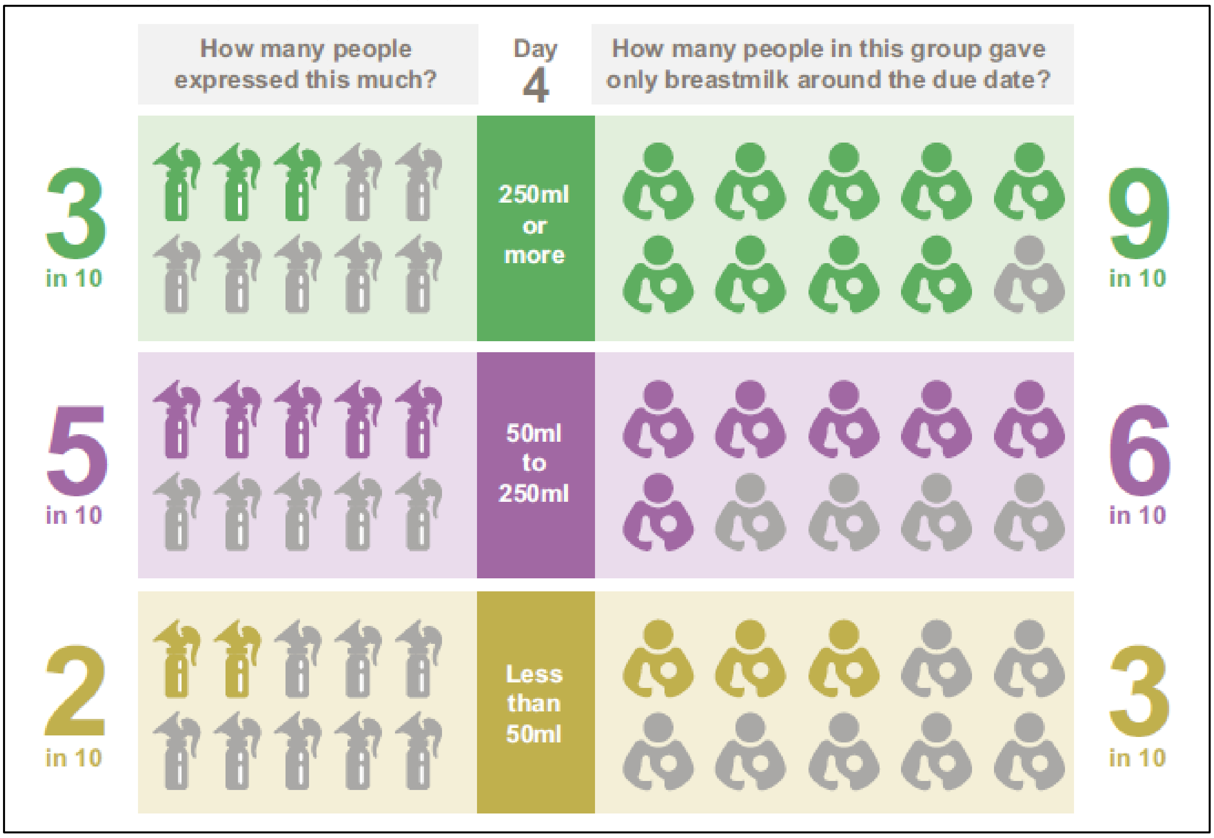

Supplementary Figure 5: Infographic for parents (day 21)

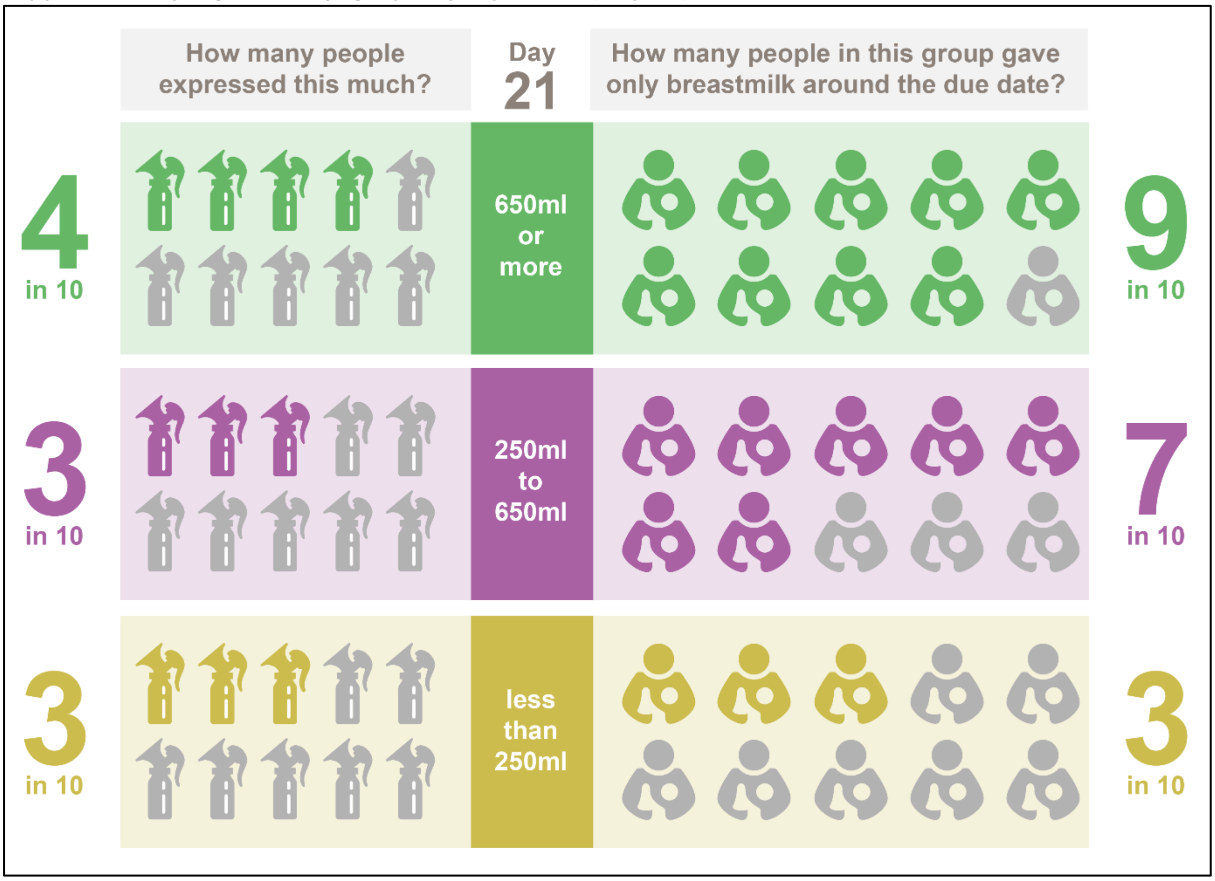

Supplementary Figure 6: Milk yield according to participant perception of milk supply, day 4 and 21

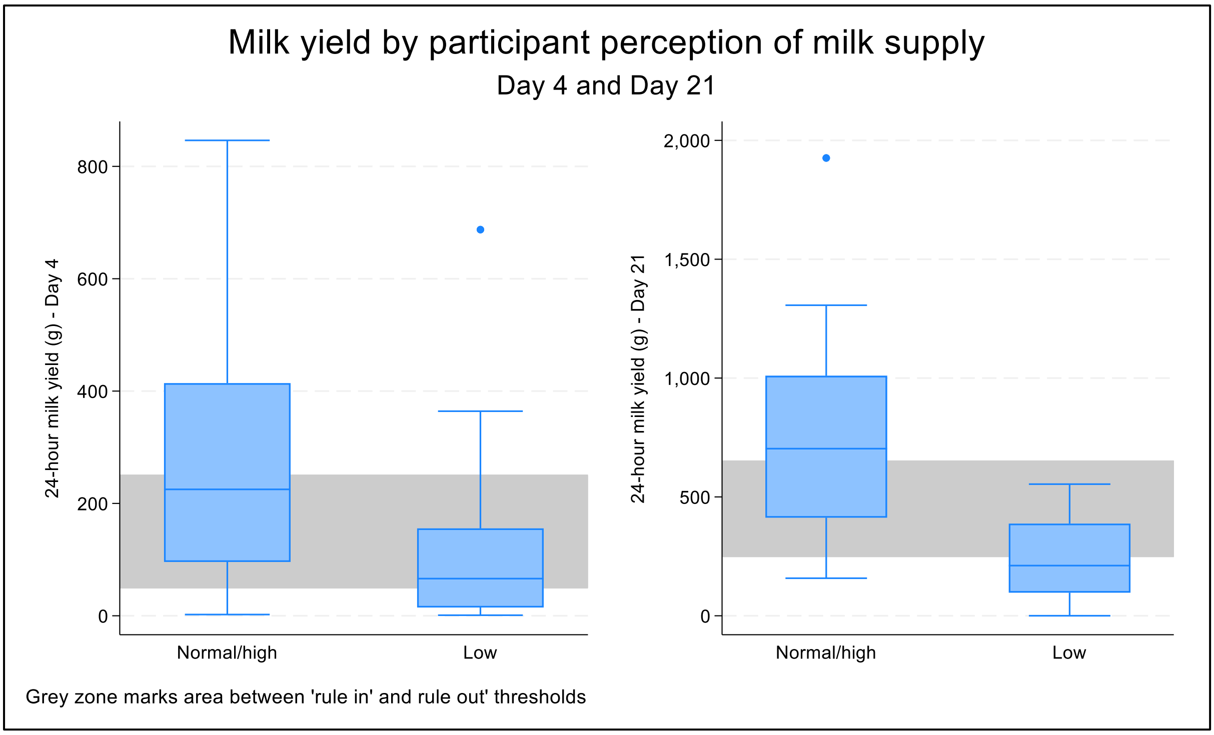

Supplementary Table 1: Comparing potential 'rule in' milk yield thresholds at day 4 and day 21.

	Sensitivity	Specificity	PPV	LR+	Sensitivity	Specificity	PPV	LR+
	**Exclusive MOM at 36 weeks' PMA**				**Exclusive MOM at two-months' CA**			
**Day 4**								
250g	29/61 (47.5%)	**28/32 (87.5%)**	29/33 (87.9%)	3.8	6/10 (60%)	**36/44 (81.8%)**	**6/14 (42.9%)**	**3.3**
200g	38/61 (62.3%)	27/32 (84.4%)	**38/43 (88.4%)**	**4.0**	7/10 (70%)	29/44 (65.9%)	7/22 (31.8%)	2.1
150g	45/61 (73.8%)	26/32 (81.3%)	45/51 (88.2%)	3.9	8/10 (80%)	25/44 (56.8%)	8/27 (29.6%)	1.9
**Day 21**								
750g	24/58 (41.4%)	**22/25 (88.0%)**	24/27 (88.9%)	3.5	7/11 (63.6%)	**29/35 (82.9%)**	**7/13 (53.9%)**	3.7
650g	32/58 (55.2%)	**22/25 (88.0%)**	32/35 (91.4%)	4.6	8/11 (72.7%)	**27/35 (82.9%)**	8/16 (50.0%)	**4.2**
600g	33/58 (56.9%)	**22/25 (88.0%)**	**33/36 (91.7%)**	**4.7**	8/11 (72.7%)	27/35 (77.1%)	8/16 (50.0%)	3.2
550g	36/58 (62.1%)	20/25 (80.0%)	36/41 (87.8%)	3.0	9/11 (81.8%)	24/35 (68.6%)	9/20 (45.0%)	2.6
500g	37/58 (63.8%)	19/25 (76.0%)	37/43 (86.1%)	2.7	9/11 (81.8%)	24/35 (68.6%)	9/20 (45.0%)	2.6

Best performing yields are marked in bold. Proposed chosen thresholds are shown in grey. PPV = positive predictive value. LR+ = likelihood ratio of the outcome when the threshold is exceeded

Supplementary Table 2: Comparing potential 'rule out' thresholds at day 4 and day 21.

	Specificity	Sensitivity	NPV	LR-	Specificity	Sensitivity	NPV	LR-
	**Exclusive MOM at 36 weeks' PMA**				**Exclusive MOM at two-months' CA**			
**Day 4**								
150g	26/32 (81.3%)	45/61 (73.8%)	26/42 (61.9%)	0.32	25/44 (56.8%)	8/10 (80%)	25/27 (92.6%)	0.35
50g	13/32 (40.6%)	**56/61 (91.8%)**	**13/18 (72.2%)**	**0.20**	13/44 (29.6%)	**9/10 (90%)**	13/14 (92.9%)	0.34
No lactogenesis II	19/32 (59.4%)	53/61 (86.9%)	19/27 (70.4%)	0.22	19/44 (43.2%)	**9/10 (90%)**	**19/20 (95%)**	**0.23**
**Day 21**								
250g	14/25 (56%)	52/58 (89.7%)	**14/20 (70%)**	**0.18**	36/44 (81.8%)	6/10 (60%)	36/40 (90%)	**0.31**
200g	9/25 (36%)	**54/58 (93.1%)**	9/13 (69.2%)	0.19	29/44 (65.9%)	**7/10 (70%)**	**29/32 (90.6%)**	0.46

Best performing yields are marked in bold. Proposed chosen thresholds are shown in grey. NPV = negative predictive value. LR- = likelihood ratio of the outcome when yield is below the threshold
